# *Leishmania* 360°: Guidelines for Exosomal Research

**DOI:** 10.3390/microorganisms9102081

**Published:** 2021-10-02

**Authors:** Áurea Martins Gabriel, Adan Galué-Parra, Washington Luiz Assunção Pereira, Ketil Winther Pedersen, Edilene Oliveira da Silva

**Affiliations:** 1 Global Health and Tropical Medicine, GHTM, Institute of Hygiene and Tropical Medicine of NOVA University of Lisbon, IHMT-UNL, 1349-008 Lisbon, Portugal; 2 Laboratory of Structural Biology of Institute of Biological Sciences of Federal University of Pará, Av. Augusto Correa 01, Belém 66075-110, PA, Brazil; adangalue@gmail.com (A.G.-P.); edilene@ufpa.br (E.O.d.S.); 3 Laboratory Animal Pathology of Federal Rural University of the Amazon, UFRA, Belém 66077-830, PA, Brazil; wkarton@terra.com.br; 4 Thermo Fisher Scientific AS Postboks 114, Smestad, 0309 Oslo, Norway; ketil.pedersen@thermofisher.com; 5 National Institute of Science and Technology in Structural Biology and Bioimaging, UFRJ, Rio de Janeiro 21941-902, RJ, Brazil

**Keywords:** isolation and description of exosomes, exosomal research guidelines, intercellular communication and host manipulation, *Leishmania* extracellular vesicle cargo, leishmaniasis

## Abstract

*Leishmania* parasites are a group of kinetoplastid pathogens that cause a variety of clinical disorders while maintaining cell communication by secreting extracellular vesicles. Emerging technologies have been adapted for the study of *Leishmania*-host cell interactions, to enable the broad-scale analysis of the extracellular vesicles of this parasite. *Leishmania* extracellular vesicles (*L*EVs) are spheroidal nanoparticles of polydispersed suspensions surrounded by a layer of lipid membrane. Although *L*EVs have attracted increasing attention from researchers, many aspects of their biology remain unclear, including their bioavailability and function in the complex molecular mechanisms of pathogenesis. Given the importance of *L*EVs in the parasite-host interaction, and in the parasite-parasite relationships that have emerged during the evolutionary history of these organisms, the present review provides an overview of the available data on *Leishmania*, and formulates guidelines for *L*EV research. We conclude by reporting direct methods for the isolation of specific *L*EVs from the culture supernatant of the promastigotes and amastigotes that are suitable for a range of different downstream applications, which increases the compatibility and reproducibility of the approach for the establishment of optimal and comparable isolation conditions and the complete characterization of the *L*EV, as well as the critical immunomodulatory events triggered by this important group of parasites.

## 1. Introduction

**Figure 1:**
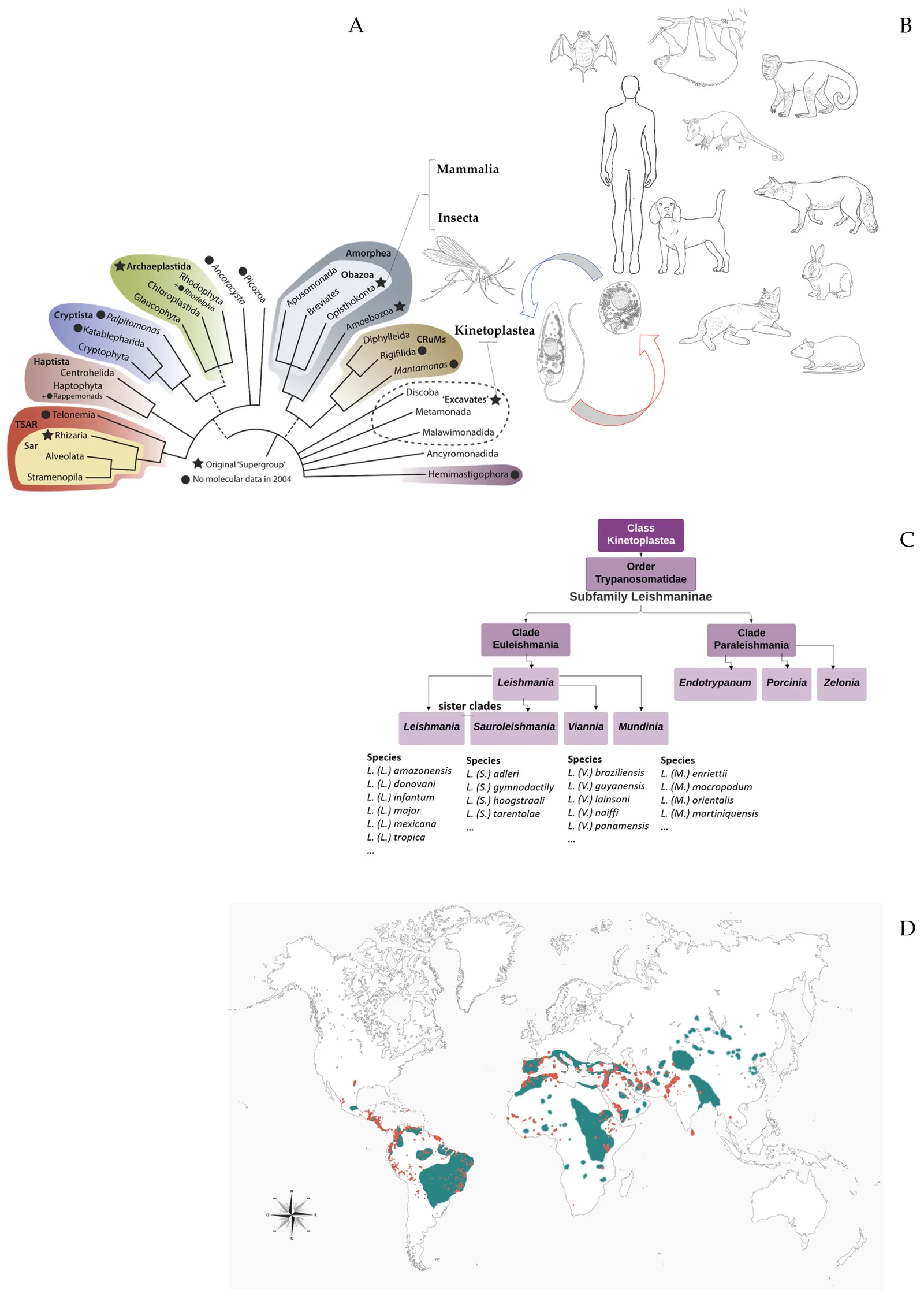
Leishmaniasis (a group of vector-borne zoonotic diseases): parasites, hosts, and epidemiological map. (**A**) Eukaryote evolutionary tree focusing on the supergroup Opisthokonta: more than 90 phlebotomine species (Class Insecta) can transmit *Leishmania* to vertebrate hosts of the class Mammalia during their opportunistic and eclectic feeding behavior; and the largest assemblage of protists of the Excavata (Kinetoplastea) [9,20,21,22]. (**B**) Zoonotic cycle of *Leishmania* [20]. (**C**) Leishmanine parasites: more than 20 *Leishmania* species have the potential to be transiently infectious in humans and the taxonomy of the dixenous genus *Leishmania* related taxonomically to the heteroxenous trypanosomatid genera of the subfamily Leishmaninae [23,24,25,26,27]. (**D**) Tracking the global distribution of endemic areas in the tropics, subtropics, and southern Europe: (1) cutaneous leishmaniasis (red dots); (2) visceral leishmaniasis (green areas). Both forms present expanding geographic ranges and rapid adaptation influenced by risk factors, with new epidemiological scenarios emerging in previously disease-free areas [9,28,29].

**Figure 2:**
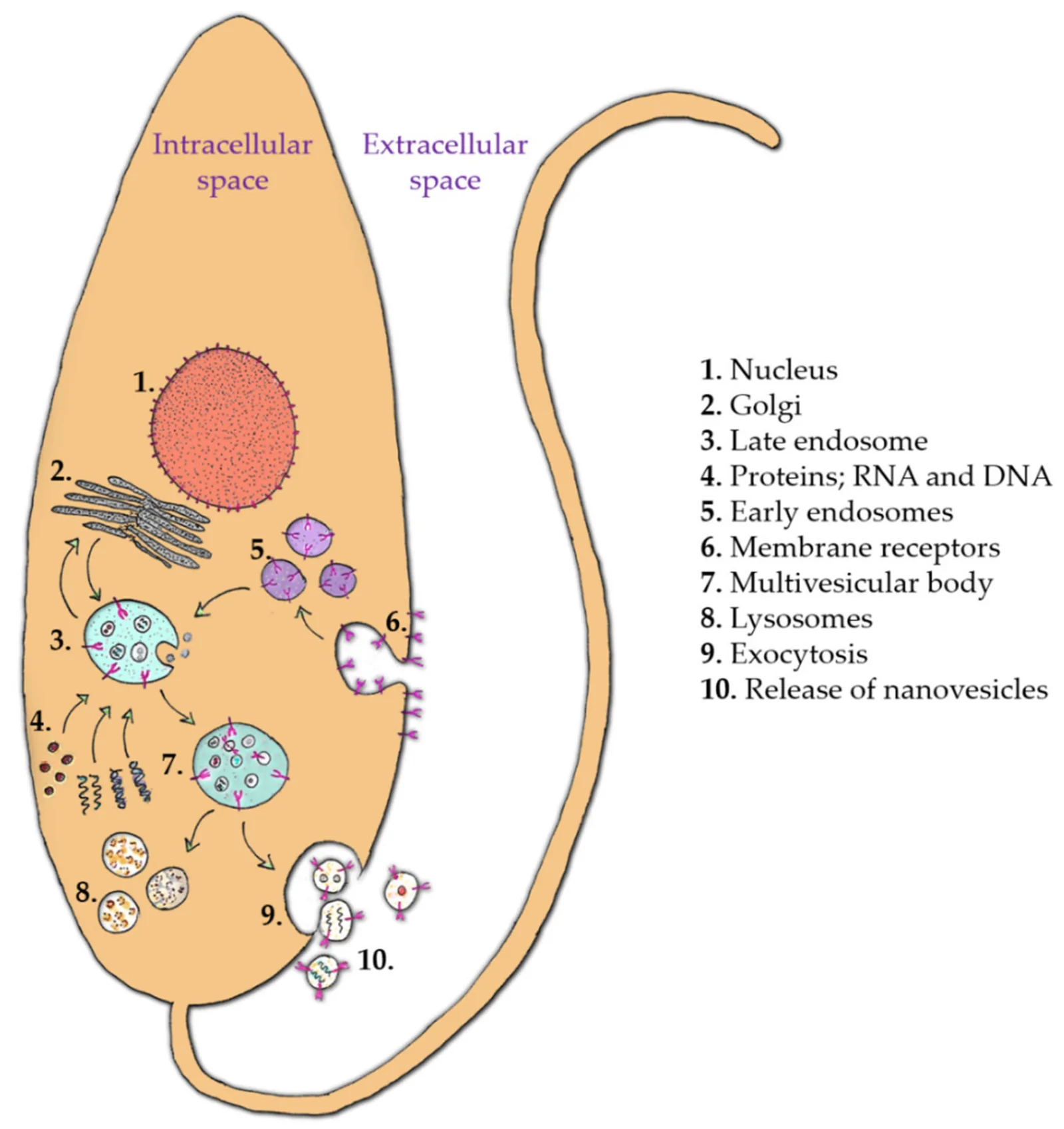
Biogenesis and release of the *Leishmania* exosomes and vesicle cargo. Diagram showing the development of the nanovesicle, which begins with endocytosis, forming the early endosomes, which develop into late endosomes by inward budding, and finally multivesicular bodies that may either undergo degradation (generating lysosomes) or merge with the cell membrane and, by exocytosis, release intraluminal endosomal vesicles that become exosomes in the extracellular environment [30].

Parasites from the subfamily Leishmaniinae of medical and veterinary importance establish infection by releasing heterogeneous extracellular vesicles that carry large amounts of molecules (Figure 1) [1,2,3,4]. These hemoflagellates have a high level of genetic variability in vivo and a propensity for rapid evolution in culture medium, which supports the broad-spectrum modulation of host immunity through extracellular vesicular communication, and enables these organisms to parasitize an enormous diversity of hosts, as well as being transiently infectious in humans (Figure 1) [4,5,6,7]. This ability to infect multiple species has facilitated the spread of *Leishmania* worldwide, and despite the development of some veterinary vaccines, the lack of effective vaccines and drug treatments for humans has allowed the rate of infections to continue to increase [8,9,10,11]. Promising new technology, including nanotechnological approaches, have developed over the past few decades to improve the prevention of leishmaniasis, the detection of the parasite, and its treatment [8,11,12,13]. The importance of the extracellular vesicles has stimulated considerable interest in the scientific community, given their apparent potential for the development of effective diagnostic and therapeutic approaches, including the prediction of the outcome of the interaction between cells [14,15,16,17]. Despite some advances, the mechanisms of the selective packaging of these extracellular vesicles are still poorly understood, and there is no consensus on the differential isolation and characterization of the extracellular vesicles or the ultrasensitive detection of specific extracellular vesicle subtypes, their specific biomarkers or their biogenesis (Figure 2) [5,16,17,18,19]. Given these considerations, we present an overview of this broad approach, with emphasis on the extracellular activity of the parasites of the genus *Leishmania*. This study will provide a reference base for future applied nanotechnological research toward the control and treatment of visceral, cutaneous, and muco-cutaneous leishmaniasis.

## 2. *Leishmania* (Kinetoplastida: Trypanosomatidae): Eclectic Parasites That Modulate the Host Immune Response through Extracellular Communication

Extracellular vesicles may be released directly from the parasite organellar compartments or through host cells infected by *Leishmania* or stimulated by antigens in response to in vitro or in vivo physiological stressors, as well as following exposure to activation stimuli [8,9,10,11,12,13,14,15,16,17,18,19,20,21,22,23,24,25,26,27,28,29,30,31].

### 2.1. Leishmanine Trypanosomatids Are Unicellular Organisms and Excellent Models to Explain Microbial Virulence

Over time, *Leishmania* parasites have developed a number of highly effective strategies to overcome specific protective mechanisms by modulating the physiological responses of different host immune systems and signaling pathways, as well as the secretion of virulence factors [14,32,33,34,35]. The *Leishmania* virulence factors include lipophosphoglycan (LPG), surface acid proteinase (GP63), glycoinositolphospholipids (GIPLs), proteophosphoglycan (PPG), A2 protein, the kinetoplastid membrane protein (KMP-11), nucleotidases, heat-shock proteins (HSPs), and transmembrane transporters, which support the survival and propagation of the parasite in the host cell [14,32,33,34,35]. The external surface of the plasma membrane of leishmanine trypanosomatids has a dense glycocalyx composed of a number of different molecules, including LPGs, PPGs, glycoprotein 63, and GIPLs, which are anchored by a glycosylphosphatidylinositol (GPI) [14,32,33,34,35]. The biochemistry and cell biology of *Leishmania* are basically very similar to those of other kinetoplastids, which present distinct population-specific genetic variation and differentiation associated with each type of proliferative cell (stage-specific differential gene expression) and transmission cell [36,37,38,39,40].

**Figure 3:**
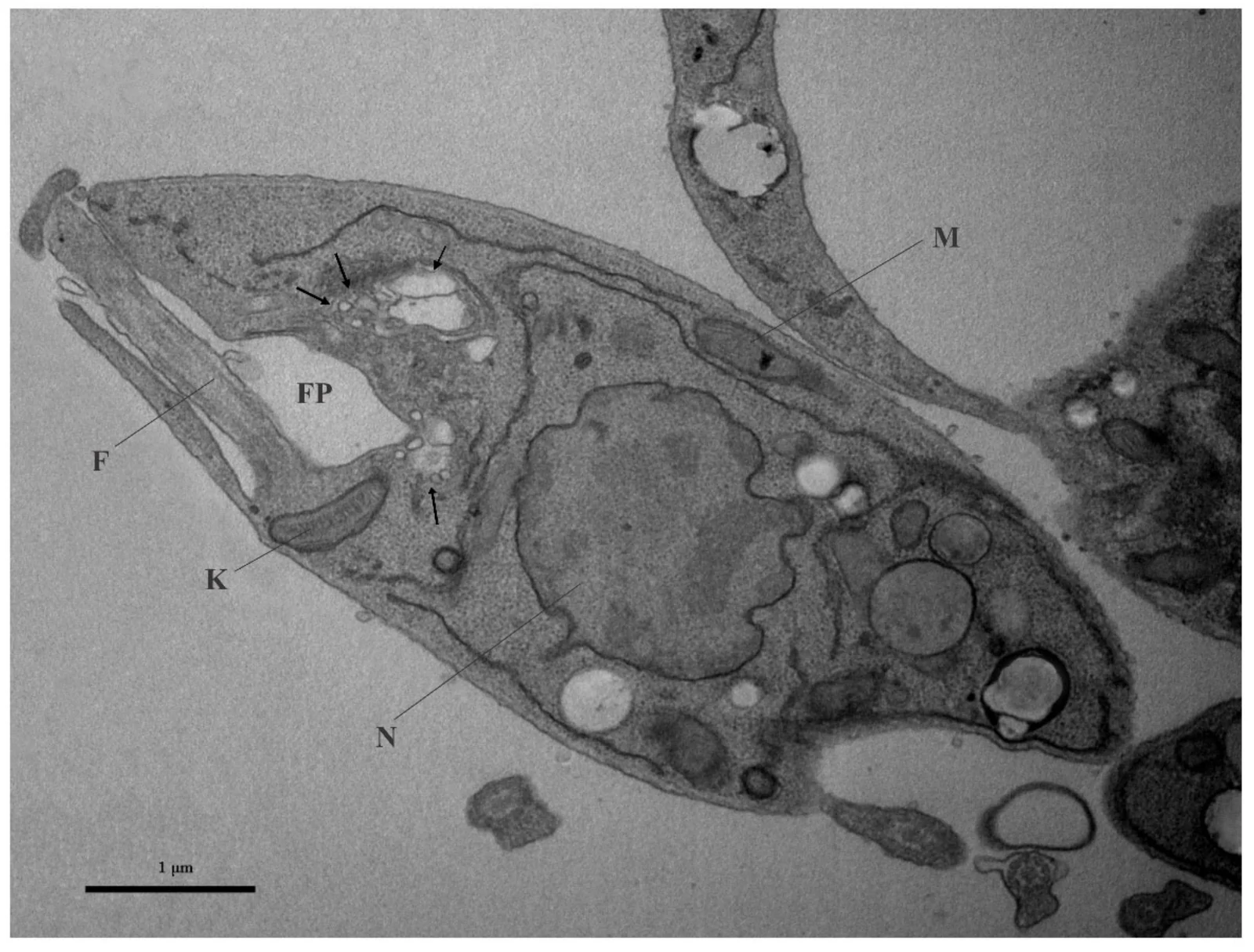
The tubular structures and vesicles (arrows) that participate in the processes of endocytosis and exocytosis can be seen in the flagellar pocket of the *Leishmania* (*L*.) *amazonensis* promastigotes (MHOM/BR/2009/M26361 strain). F = flagellum; K = kinetoplast; M = mitochondrion; N = nucleus. Scale bar = 1 µm.

These parasites have a digenetic life cycle adapted to their specific hosts and vectors, together with a variety of developmental forms defined by cross-linked sub-pellicular corset microtubules covalently linked to the plasma membrane and covering the whole cell [41,42,43,44]. Cell division depends on the insertion and elongation of these microtubules into the existing array interacting with microtubule-associated proteins, microtubule-severing factors and kinesins influencing, for example, modifications of the cytoskeleton, such as cell rounding and a decrease in flagellum length, during *Leishmania* cell death [41,42,43,44]. In vitro studies of the differentiation of *Leishmania* (*Leishmania*) *amazonensis* amastigotes into promastigotes have used Electron Microscopy (EM) to define these characteristic arrays [45]. At present, caution is required defining cell types based solely on their morphology, given that few molecular markers are available to identify life cycle forms [35,38,46,47]. The shape and configuration of the *Leishmania* cell are nevertheless linked intimately with the pathogenicity and ecological niche of the species, and must be transmitted accurately from one generation to the next during mitotic division, although recent studies have shown meiosis-like sexual recombination in *Leishmania*, and other sexual processes may be possible [22,41,48,49,50]. Both forms of development conserve their basic cell architecture and ultrastructural features over the course of the life cycle, with the kinetoplast anterior to the nucleus [44,45,47,49,50]. In these cells, much of the single, branched mitochondrion contains kinetoplast DNA (kDNA) that is connected to the basal body, where a single flagellum extends from the flagellar pocket (FP), to which it is attached by a cytoskeletal structure called the Flagellum Attachment Zone (FAZ) (Figure 3) [40,41,44,45,46]. Up to now, the flagellar pocket of *Leishmania* has been identified as the only site of endocytosis and exocytosis (Figure 3). Thus, during the parasite lyfe cicle the FAZ is a crucial feature playing an important role in the resolution of cell membrane organization and morphogenetic resolution of the anterior extremity of the parasite cell during successful *Leishmania* cell division (cytokinesis) [41,44,46]. In addition, in vitro studies have highlighted the influence of the morphological parameters of the different *Leishmania* developmental forms (promastigote and amastigote), especially when they are exposed to specific cell-extracellular matrix (ECM) interactions and maintain specific vital functions that enable their survival during host infection [40,41,44,45,46]. All this variation emphasizes the need for the identification of new, independent markers of the different cell types of the *Leishmania* life cycle, such as the procyclic and metacyclic promastigotes identified in the sand flies, based on the dimensions of the cell body and flagellum [41,44,46]. Following infection, the parasites interact with the phagocytic cells of the mammalian host, replicating within compartments enclosed by membranes and establishing a persistent infection by inducing macrophage dysfunction through the direct manipulation of macrophage signaling, which may be initiated by the flagellum, but involves predominantly the cell body [39,41,44,46]. In the infected macrophages, the promastigotes shift to amastigote form, which changes the shape of the cell, to minimize its surface area to volume ratio, which reduces the area exposed to the harsh environment of the different types of parasitophorous vacuole, according to the specific requirements of each *Leishmania* species [51].

Further studies will thus be necessary in order to elucidate the role of the cellular components in its organization and their relationship with the molecular cargo and the surface receptors used in target cell recognition over the course of the *Leishmania* life cycle [52,53]. Overall then, the different developmental forms of *Leishmania* can be distinguished by their morphology, environmental and nutritional requirements, and activatory stimuli *in vitro*, as well as their metabolism, motility, growth rate, and multiplication pattern in the host or in culture medium, as well as the molecular expression regulated by extracellular vesicles [54].

### 2.2. Host Leishmania Interactions through Extracellular Products

Although infection does not necessarily generate symptoms, some species of *Leishmania* are multi-host pathogens that cause disease in both humans and other mammals, such as dogs (*Canis lupus familiaris*) which are important reservoirs of *Leishmania*. Specifically, *Leishmania* infection is being associated with a determined pathology and its typical histopathological and clinical features, suggesting that the extracellular vesicles released by *Leishmania (*internalized by phagocytic cells and transformed into the obligate intracellular amastigote form) can contribute to the host immunomodulation and favors parasite survival and disease progression [20,26,55,56]. For comparison purposes, the phenotypic variation of the susceptibility of dogs to *Leishmania* (*L*.) *infantum* may be a consequence of the activity of the genetic markers that control both pro- and anti-inflammatory cytokines (immunomodulatory activity during leishmaniasis pathogenesis) and the different patterns of the cellular immune response to the presence of *Leishmania* [57,58,59]. We observe that clinical manifestations in dogs are quite extremely variable and represent an association of visceral and skin disease, serving as an animal model for different hypotheses formulated about the molecular expression and the roles of parasite-derived extracellular vesicles.

There is increasing evidence that the prolonged persistence of *Leishmania* in infected patients is a frequent phenomenon, which leads to the development of immunocompromised individuals and, in some cases, immunosuppression which affects the mechanisms of the innate and adaptive immune systems, leading to an inefficient and polarized immune response [14,20,34,41,60]. According to Gabriel et al. (2019), cutaneous leishmaniasis has an ample geographic distribution and polymorphic clinical features, with the competence of innate and acquired immune responses determining the outcome of infection and the severity of the cutaneous leishmaniasis [20]. Despite the many advances in clinical studies and our ever-growing knowledge of leishmaniasis, the spread of these parasitic diseases and the specific details of their host-parasite interactions are still not fully understood [20,56,61,62]. This makes it difficult to fully understand the dynamics of the course and severity of *Leishmania* infection, and the persistence of the host-pathogen relationship throughout the course of the disease in the host [20,56,61,62].

*Leishmania*-host interactions are considered to be excellent models of virulence factors, the efficiency of survival and transmission strategies over the life cycle, the ability to interact with the physiology and complex molecular mechanisms of the host cell, the modification of the cellular environment, the establishment of different niches, and modulation with the histopathological and immune responses of different hosts [20,38,63,64]. The uptake of the promastigotes by the phagocytes involves a number of different strategies that enable the parasite to interfere with the sequential protective actions of the two principal host immunological mechanisms [20]. Following infection of the skin, the immunological response of the host to *Leishmania* is characterized initially by the innate immune response, during the primary stage of the infection, followed by the adaptive immune response [20]. The adaptive mechanisms are linked by antigen-producing cells, such as the dendritic cells, and by cytokines released into the microenvironment by the effector immune cells [20]. The control of the dendritic cells and the activation of the lymphocytes lead to the secretion of the pro-inflammatory cytokines IL-2, IFN-γ and TNF-α, which can activate the microbicidal mechanisms of the macrophages, leading to the inactivation of the parasite, and the activation of the cytokines IL-4, IL-5 and IL-13, which permits the establishment of the disease [20]. During cutaneous *Leishmania* infection and its complex subset of interactions, the differentiation of the Th17 lymphocytes sustains a strong inflammatory environment, which is associated with the persistence of the lesion and is characterized by the pro-inflammatory modulator IL-17 [20]. This modulator induces other cells to release inflammatory mediators that ultimately promote the recruitment of Polymorphonuclear neutrophils (PMNs) to the infection site, which favors the progression of the disease [20]. On the other hand, visceral leishmaniasis in naturally infected dogs may present an increased frequency of T lymphocytes, in particular CD8 + T cells, increased regulation of MHC-II expression (in higher parasite loads) by the lymphocytes, and decreased levels of CD21 + B cells [1,20,65,66,67]. However, much remains to be learned about the mechanisms that *Leishmania* uses to influence the target host, signaling pathways and how they contribute to deactivate the phenotype seen in *Leishmania*-infected macrophage. Thus, considering the importance of dogs (the major hosts for *Leishmania* parasites and the main reservoir host for human infection), actually the author Á. Gabriel is studying the immunomodulatory effects of *Leishmania* extracellular vesicles (*L*EVs) in dog host cells, against the background of the project “EXOTRYPANO Achieving new frontiers through trypanosomatid exosomes (TEx).

Advances in the understanding of the progression of leishmaniasis indicate that the cellular interactions are more complex than the simple Th1/Th2 dichotomy, and may depend on the degree of humoral immunity. High levels of IgG predict the persistence of the parasite and are regulated by the extracellular vesicles produced by both immune and non-immune cells [14,20,66,67]. The identification of specific cargo molecules in the *L*EVs indicate that they have an adjuvant-like function in the immune response, which may infer a key advantage for the establishment of the infection and progression of the disease, which induces both quantitative and qualitative changes in the protein content of the infected host cells and the extracellular vesicles [14]. In this case, the proteinases of both the host (matrix metalloproteinases) and parasite (cysteine proteinases, metalloproteinases, and serine proteinases) affect the dynamics of the *Leishmania* infection [66,67]. These in vitro advances hint at a potential, previously unrecognized mechanism of *Leishmania* pathogenesis mediated by the *Leishmania* extracellular vesicles (*L*EVs) in the complex analysis of the dynamic molecular mechanisms of the *Leishmania* life cycle [5,68,69]. This indicates that the *L*EVs may play a significant role in the pathogenesis of a number of different parasite species, which hints at the potential therapeutic use of these substances in some cases [5,68,69]. The integrated understanding of these parasites (in the different clinical forms of leishmaniasis) and their activity patterns in their different forms of development and hosts is an ample field of research that reinforces the need to study the different species and the specific *L*EV pathways of their extracellular production in more detail.

## 3. *Leishmania* Extracellular Vesicles (*L*EVs): Do We Know Everything?

By using these intrinsic *L*EVs, *Leishmania* parasites retained sophisticated survival pathways in their most ancient evolution [70,71,72,73,74]. Overall, *L*EVs are heterogeneous, membrane-limited structures that play important roles in numerous biological processes, both pathophysiological and physiological, and in specific intercellular crosstalk between cells, transferring information through multiple cargoes and modulating the immune system of the host [70,71,72,75,76].

### 3.1. Trypanosomatids Extracellular Vesicles Studies Requirements

**Figure 4:**
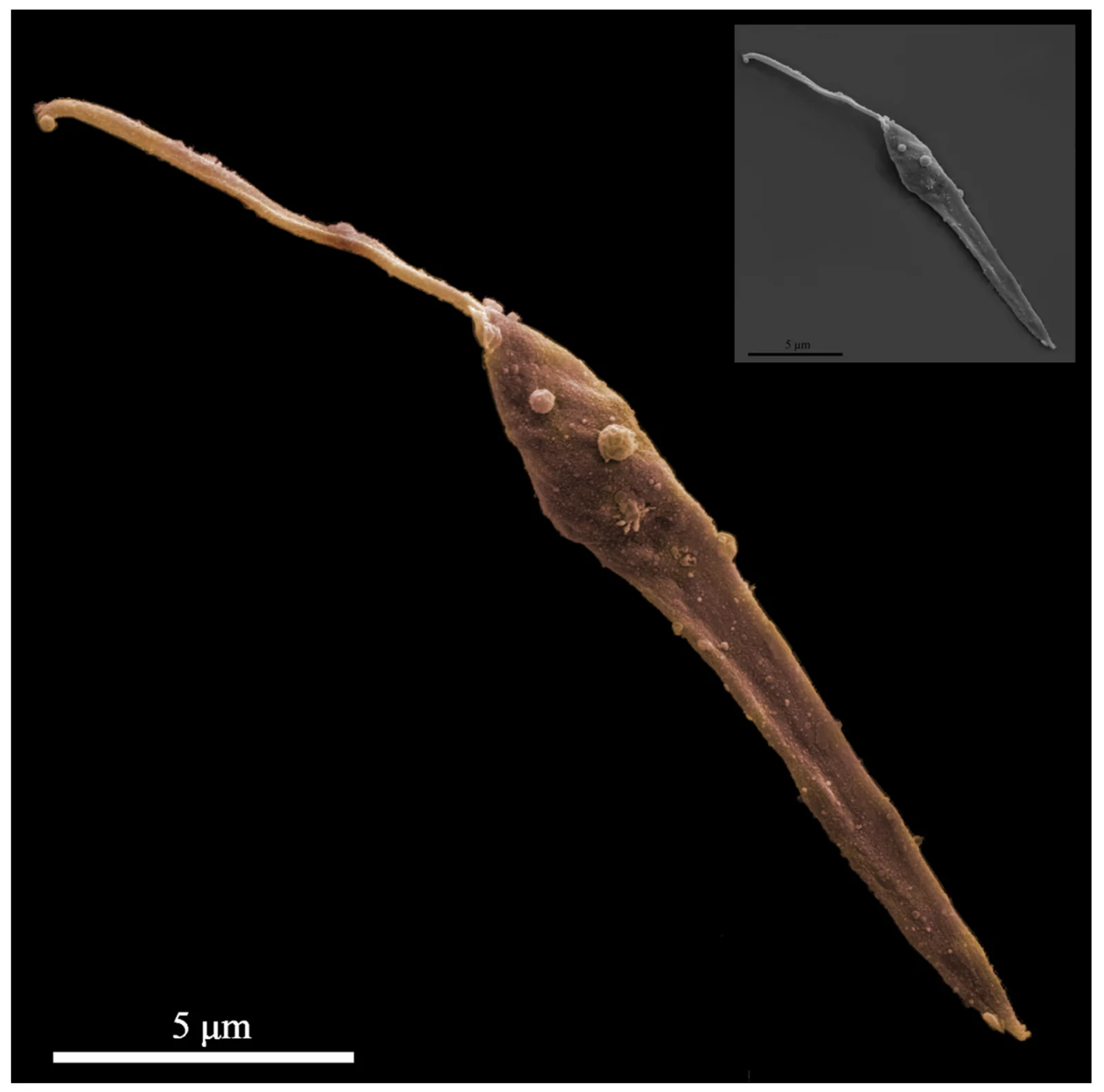
Heterogeneous group of membrane-bound extracellular vesicles secreted by *Leishmania*. Representative SEM photomicrograph showing surface membrane vesicular forms (light points, rounded and amorphous vesicles) of *L.* (*L*.) *amazonensis* promastigotes (MHOM/BR/2009/M26361 strain). Scale-bar 5 µm.

In the present review, the term “*Leishmania* extracellular vesicles” (*L*EVs) is used to refer to the naturally released spheroidal nanoparticles of polydispersed suspension that are surrounded by a lipid layer of the parasite membrane [77,78] (Figure 4). As in prokaryotes, higher eukaryotes, and other trypanosomatids, TriTryps (*Trypanosoma Schizotrypanum* *cruzi*, *T. Trypanozoon brucei* and *L. L. major*) may produce extracellular vesicles (exosomes or exosome-like vesicles) and secrete proteins into different extracellular environments, which are transported in vesicles through a selective and efficient pathway from the endoplasmic reticulum (ER) to the Golgi apparatus and the FP [14,44,78]. Scientists increasingly study the primary role of the structure and composition of the TriTryps extracellular vesicles in order to better understand the role of these molecules in the parasitism process [78]. From this perspective, a number of different approaches can help to better understand the role of the extracellular vesicles of the parasite, including insights from in vitro studies, which are essential to biomedical research and comparative scanning electron microscope studies of *Leishmania* nanovesicles [77,78]. While trypanosomatid studies must clearly be standardized, in terms of their generic parameters, such as the temperature, which affects the regulation of the expression of genes coding for the proteins involved in the metabolism of lipids and carbohydrates, the avoidance of oxidative stress, and the processes of proteolysis and phosphorylation, as well as the decrease of the ribosomal proteins involved in translation [79]. In the artificial environment, the temperature shift (ambient temperatures of 25–26 °C and 37 °C) is an important factor influencing the secretion of proteins via exosome-like vesicles on the surface of the cultured *Leishmania* parasites during their replication and development into metacyclic promastigotes, which probably affects the levels of this extracellular production [80,81].

### 3.2. Current and Expected Advances in Leishmania Extracellular Vesicle Research

In recent years, research efforts have focused increasingly on the development of experimental approaches to the study of the parasitic extracellular vesicles, in response to the recent expansion of interest in the role of *L*EVs in the pathogenesis of both cutaneous and visceral leishmaniasis [2,6,82,83,84]. Emerging technologies such as proteomic and bioinformatic analyses have been adapted for the study of the extracellular vesicles released from parasites in vitro [3,85,86,87,88]. To further our understanding of the *Leishmania*-host-cell interactions, a broad-scale analysis of the cargo of their production of extracellular molecules has created very promising perspectives for the development of innovative applications [3,85,86,87,88]. In this context, the implementation of standardized isolation and analysis techniques for the extracellular vesicles, supported by bioinformatic and biostatistic expertise for data processing and analysis, has highlighted their potential as clinical biomarkers for leishmaniasis and monitoring, and as therapeutic agents [89].

**Figure 5:**
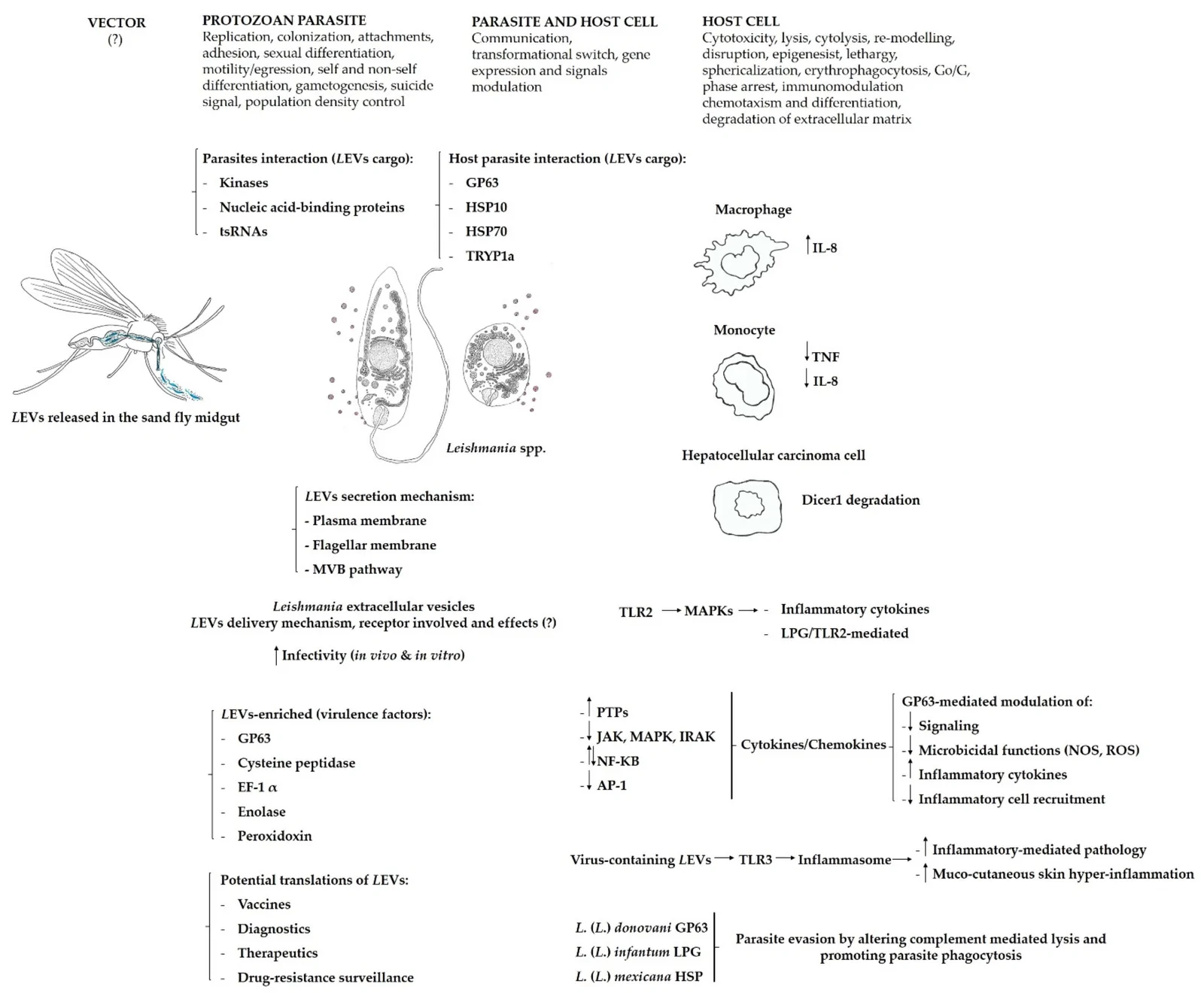
Extracellular vesicles as vehicles between *Leishmania* and host cell communication [5,8,76,85].

In general, *L*EVs are exported by either classical or non-classical molecular mechanisms and are involved in the transfer of biologically active molecules, including proteins, lipids, metabolites, miRNAs, and nucleic acids [86,90,91]. Although their role in the infectivity and development of *Leishmania* is still poorly understood, there is irrefutable evidence that *L*EVs have important functions, which require further research to clarify aspects such as their molecular pathways and host cells interactions (Figure 5) [8,14,92,93,94]. The extracellular vesicles effector cargo is known to be delivered into the host target cells, stimulating both pro- and anti-inflammatory immune responses [14,40,95,96,97]. These vesicles have been implicated in phenotypic changes of the cell and in intracellular communication, such as the delivery of antigens and the transport of macromolecular messages of proteins and nucleic acids with regulatory potential, as well as the removal of unwanted molecular components for cell maintenance [2,7,15,98,99]. Other functions attributed to *L*EVs include the export of proteins, their response to environmental change, and the modulation of the response of the cytokines [1,3,4,40,100]. Concomitant advances in mass spectrometry have contributed to the identification of complex proteins and the accurate analysis of the segregation of the secretome by the living cells (microscopic unicellular and multicellular organisms) into the extracellular space, which includes adhesion molecules, chemokines, cytokines, enzymes, and other factors, which has been applied successfully to the discovery of protein biomarkers in the extracellular vesicles [67,101,102,103,104]. The available data on isolated *L*EV subpopulations strongly suggest that these vesicles represent the future of biomarkers in medical Parasitology or that they are the information vectors able to modify the range of the genes expressed within the recipient cells [18,60,89,105,106]. Based on recent advances, expectations for future research include the development of alternative methods for the isolation of *L*EVs, and new techniques for the rapid assessment of single *L*EVs subpopulations. From our experience, we would strongly encourage the comparative analysis of different strains and advances in future research on integrated metabolomics and the analysis of the proteomics of these subpopulations.

### 3.3. Leishmania Extracellular Vesicles: What We Know So Far

Since 2007, there has been a growing number of experimental in vitro and in silico studies of the role of *L*EVs, as well as studies of the extracellular vesicles of other pathogenic unicellular eukaryotes, including microaerophilic extracellular protozoan parasites, in relation to their biogenetic pathways and infection strategies [14,76,82,94,107]. These studies have focused on the following points: (1) through which mechanisms the parasites generate extracellular vesicles that they transfer to a wide range of molecules; (2) how the extracellular vesicles are used as vehicles for cell signaling and the manipulation of the host’s immune system, and (3) how they elicit the pro-inflammatory response that causes disease, which plays a key role in macrophage modulation [108,109,110,111,112]. Given the different *Leishmania* morphotypes, we would expect future studies to shed light on the molecular factors involved in the pathogenesis of *Leishmania*, evaluating the effects of the *L*EVs on the immune response of the recipient host [1,2,14,76,84]. Exosomes of *L.* (*L.*) *infantum chagasi* in the procyclic and stationary (metacyclic-like) phases have discrete protein profiles in which approximately 50 virulence factors have been detected including molecules for immunomodulation and evasion (GP63, EF1α, Oligopeptidase), increased pathogenicity (Casein kinase, KMP-11, Cysteine Peptidase and BiP), and parasite protection (Peroxidoxin) [35]. In particular, GP63 is known to be significantly down-regulated and to shift its location in the parasite as promastigotes are transformed into amastigotes in the infected macrophages [34]. Given this shift in the location of the GP63 within the parasite, it is unclear whether the later stage macrophage infections, which harbor the amastigotes, continue to release GP63 into the exosomes [4,5,34,40,81], and it is not known whether the parasite molecules synthesized in the amastigotes found in the macrophages in long-term infections are released into the exosomes [35,40,54,68].

The vast majority of published *L*EVs studies of different species have shown that a low percentage (5–9%) of exosomal proteins contain a signal peptide, which is strikingly similar to higher eukaryotes and their ancient, universal origins, which suggests that a majority of the proteins of the secretome are secreted non-conventionally, with no peptide signal [3,14,68,113]. A number of studies have also revealed differences in the distribution of the intra-membranous particles (integral proteins), with the aggregation of particles in specific areas of the *Leishmania* membrane reflects their composition, function, and density, as well as the evolutionary form of the parasite, with the promastigote membrane being richer in whole proteins than the amastigote membrane [34,39,41,53,114]. The FP is an invagination of the cell membrane at the base of the flagellum, whose membrane has a smaller amount of intra-membranous particles in comparison with the body of the parasite [41,46]. However, a high concentration of these proteins can be found in the membrane surrounding the FP, forming zones of flagellar adhesion [41,46,115].

The proteomic profiles obtained from the *L*EVs have been shown to be closely dependent on the manner in which these vesicles were isolated from culture medium [3,35]. The overall outcome of the in vitro models contributes to the evaluation of the modulation of the immune response of the host cells by the *L*EVs [1,4,14,35,86]. Potentially, the complete understanding of the *L*EV cargo profile that activates the macrophages and lymphocytes or, alternatively, induces the deactivation of the cell will contribute to a better understanding of the interactions established between the parasites and the host cell [14,40,54,69,82]. In particular, this will contribute to the development of effective prophylactic and therapeutic approaches, given that the *L*EVs are the most biomimetic nanovectors of a variety of molecules, including proteins, nucleic acids, and chemicals in the poorly known molecular mechanisms of the parasite that regulate the immune response of the host to favor infection and their propagation [14,75,76,85,116].

In mammals, persistent *Leishmania* infection is linked with macrophage signaling pathways that block microbicidal functions and the innate response of the host during infection [14]. In this case, the *L*EVs are effectively macrophage immunomodulators of early host inflammatory responses, although the exact mechanisms involved in this process are yet to be fully understood [6,12,35,85,117]. The immune response is a critical aspect of the infection process and the establishment of the disease through the development of protective immunity associated with the intracellular destruction of the amastigotes by the macrophages, which will depend, in turn, on the induction of an efficient cellular response through the production of cytokines such as IFNγ, interleukins (IL), and TNF-α [20]. In vitro studies have shown that the *L*EVs of *L.* (*L.*) *infantum* recruited more macrophages and dendritic cells than other extracellular products or the parasite, which reflects the functional response of basal MHC-ll and decreased CD40 and CD 86 [8]. Many recent studies have shown that the presence of *L*EVs released from *L.* (*L.*) *donovani* modify the IFNγ-induced production of pro- or anti-inflammatory cytokines by cultured human monocytes, favoring the Th1 immune response and the elimination of *Leishmania*, which leads to the control the infection [1,69,96,118].

The T and B cells are key components of acquired immunity. The B cells are an important source of cytokines in chronic inflammatory diseases, generating antigen-specific antibodies in antibody-mediated immunity, and contributing to the activation of the T cells, which, together with the B-1 cells produce large quantities of IL-10, contributing to the persistence of the parasite and the maintenance of memory cells [20]. The in vivo analysis of the *L*EVs released from *L.* (*L.*) *amazonensis* and *L.* (*L.*) *infantum* revealed pro-inflammatory activity, which increased the parasite burden of the B-1 cells [1,3,7,100]. Given this, *L*EVs released from the promastigotes modulate the response of murine B-1 cells to *Leishmania* both in vivo and in *vitro*, although the exact mechanisms are still unclear [84]. In vitro studies have shown that *L*EVs released by both *L.* (*L.*) *infantum* and *L.* (*Viannia*) *braziliensis* induced IL-10, although they were not able to induce significant levels of pro-inflammatory cytokines in the same way that the *L*EVs released from *L.* (*L.*) *amazonensis* can elicit the production of IL-6, NO, and TNF-α [1,4,7,34,100]. The interaction of the pro-inflammatory and regulatory lymphocyte subsets appears to be a characteristic of cutaneous leishmaniasis [20]. In this case, patients infected with *L.* (*Viannia*) *braziliensis* may present CD4–CD8-T lymphocytes, which express the αβ T cell receptors associated with a more inflammatory environment, leading to the activation of antiparasitic macrophages and T cells expressing γδ T cell receptors, which play a regulatory role in the reduction of the inflammatory response [20]. In mice infected with *L.* (*L.*) *major*, the CD4+ T and B cells that healed the infection played a key role in killing *L.* (*L.*) *amazonensis* intracellular parasites [20]. Studies of mice have shown that CD4+ lymphocytes inoculated with *L*EVs presented exacerbated pathology, producing immunosuppressive cytokines IL-4 and IL-10 [1,70,80,85,119]. The Th-17 lymphocytes express interleukin IL-17A, which is associated with the progression of cutaneous leishmaniasis during *L.* (*L.*) *major* infection in murine models (i.e., neutrophil recruitment) and susceptibility during experimental visceral leishmaniasis caused by *L.* (*L.*) *donovani* [1,40,68,96,119]. Extracellular vesicles from macrophages infected with *L. (L.) amazonensis* activate the production of the pro-inflammatory cytokines IL-12, IL-1β and TNF-α by the naive macrophages, which controls the *Leishmania* infection through the Th1 immune response, while contributing to the stimulation of immune mechanisms that confer a resistant phenotype on the naive, bystander macrophages [1,7,100]. The Th1 response is characterized by elevated IL-12, IL-2, IFN-γ and TNF-α, as well as the down modulation of the production of IL-4 and IL-10, which promotes macrophage activation, a crucial control of *Leishmania* parasite burden and clinical cure [20]. In vivo Th1-Th2 responses regulate the progression of leishmaniasis, with Th2-related cytokines (IL-4, IL-5, IL-10 and IL-13) inhibiting macrophage activation, which also contributes to the survival of the parasite [20]. Experimental models have demonstrated that, in the presence of *L*EVs delivered by *L.* (*L.*) *amazonensis* promastigotes, infected animals presented a significantly higher parasite load and polarization of the Th2 response in comparison with the control group, infected with the parasite alone, which demonstrates that the *L*EVs of the *L.* (*L*.) *amazonensis* promastigotes stimulate macrophages and B-1 cells to express different types of cytokines [1]. The neutrophils also appear to have a dual effect in delaying the early establishment of infection and later favoring the pathology of the lesion, although experiments on the egestion of *L*EVs from the infectious inoculum of sand flies showed that IL-1*β* is induced through the inflammasome produced by the neutrophils, which important for the visceralization of *L.* (*L.*) *donovani*, a process that is still completely unknown [3,20,90,120]. The *L*EVs released from the promastigotes and amastigotes is a potentially ample field of research, which provides the background for comparative studies of the *L*EVs, which identified a positive selection mechanism in the poorly known mutualistic relationship of *L.* (*V*.) *guyanensis* and the LRV1 endovirus [70,72]. Ancient *Leishmania* lineages infected naturally with an endosymbiotic non-enveloped RNA virus might maximize *Leishmania* infectivity by subverting the immune response as a determinant of disease severity related to the co-circulation of the *Leishmania* viruses (LRV1 and LRV2) in the zoonotic focus on leishmaniasis in both the New and Old Worlds [70,72,73,74]. There is some evidence that parasites infected with the LRV1 virus may be more pathogenic in experimental models and in humans, exacerbating the aggressiveness of muco-cutaneous leishmaniasis [70,72,73]. The patterns of expression of the proteins of the exosomes containing LRV1 and the parasites of the Neotropical subgenus *Viannia* provide potential insights into the adaptive mechanisms in *Leishmania*-virus co-evolution, and how this affects the suitability of the parasite for viral infection and persistence [70,72]. This may explain the specific effects of the virus on the translation of the parasite mRNA during the pathogenesis of some isolates [70,72]. Research in *Leishmania* RNA virus proteomics aims to understand how capsid function and structural properties can be exploited through in vivo applications for immunization and the reduction of the clinical symptoms [71]. Despite considerable progress in this field, our understanding of the biogenesis of the *L*EVs (i.e., by the plasma membrane, canonical endosome pathways or formed during apoptosis and senescence-associated cellular processes) their subtypes, cargo and molecular shuttling mechanisms remains far from complete, and further advances will depend on the standardization of procedures and the validation of the experimental model for the more reliable comparison of results on the exosomal production of *Leishmania* (Table 1).

### 3.4. Drug-Resistant Leishmania Extracellular Vesicles

Different *L*EVs expressing a variety of virulence factors and proteins that increase virulence differentially can induce drug-resistance in parasites [1,3]. The drug-resistant *L*EVs secreted by *Leishmania* form a nucleosome with the human histones in the host chromatin during the evolution of the disease [3]. Drug-resistance mechanisms may induce changes in the morphology, size, and distribution of the *L*EVs, although, in general, they are still poorly known, and some may be non-specific adaptations that provide a general gain in fitness that allows the parasite to survive under stressful conditions [3]. Comparative studies of the *L*EVs released from the *L.* (*L.*) *donovani*, *L.* (*L.*) *major*, and *L.* (*L.*) *infantum* strains revealed resistance to antimony, miltefosine and amphotericin B, findings that contributed to further analyses with larger sets of strains and replicates, revealing their variability and potential biomarkers of *Leishmania* [3,4]. In this context, comparative studies of leishmanicidal activity can test the effectiveness of the repurposing of existing drugs, as in the case of an experimental study of buparvaquone (BPQ), a drug used for the veterinary treatment of theileriosis, which showed promising activity against *Leishmania* [69]. The in vitro development, optimization, and evaluation of the physical and chemical characteristics of nanostructured lipid carriers (NLCs) for the encapsulation of BPQ and the evaluation of its solubility compared the promastigotes of *L.* (*L.*) *amazonensis*, *L.* (*L*.) *braziliensis*, and *L.* (*L*.) *infantum* samples [69]. These findings provide a baseline for an ample field of research, in which future studies may approach all these different aspects of the clinical role of *Leishmania L*EVs, especially those of drug-resistant strains, and how they contribute to the survival of the parasite over the course of its life cycle, offering potential approaches for diagnosis, follow-up treatment, the monitoring disease progression, prognosis, and new vaccine targets [3].

## 4. Guidelines for the Production of Exosomes from Parasite Cells Grown in Culture Medium *(*Exo-Free Serum)

According to the ‘‘Minimal Information for Studies of Extracellular Vesicles’’ (MISEV2018), extracellular vesicles are a heterogeneous group of structures, which can be defined as apoptotic bodies, exosomes, and microvesicles [87,88]. Despite this, the in vivo and in vitro extracellular vesicles mechanisms of *Leishmania* remain poorly known [1,3,14].

### 4.1. Guidelines for a Hypothetical Model of Leishmania Extracellular Vesicle Research

**Figure 6:**
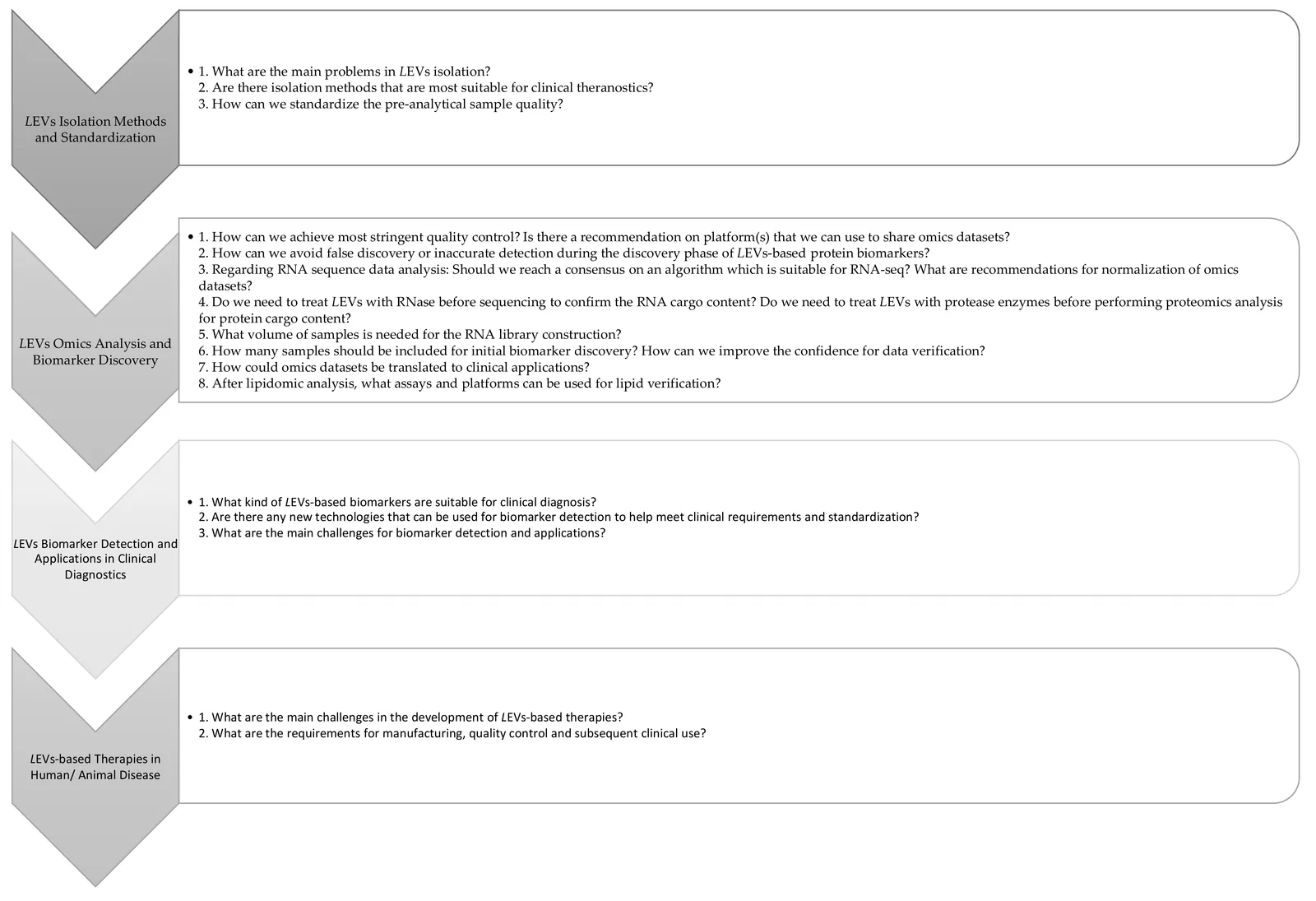
Guided reading questions for *Leishmania* extracellular vesicles research [89].

Methodological advances in the differential isolation of extracellular vesicles have supported the description of the different subtypes of the extracellular vesicle, based on their origin, size, components, and their specific or diverse functions [14,88,99,113]. The molecular and morphological evidence indicates that extracellular vesicles secreted by microbes transport a plethora of lipids, metabolites, nucleic acids and proteins, which play a prominent role in the modulation of immunity during their biogenesis (i.e., by the plasma membrane, canonical endosome pathways or formed during apoptosis and senescence-associated cellular processes) [3,14,18,89,99]. Although some hypotheses have been proposed, little is known about how these vesicles capture cell-specific proteins or how the host-derived extracellular vesicles control distinct levels of infection [3,14,18,99]. To establish a standard for *L*EV research, we also present guidelines below, which we hope can be used to orient research through both deductive and inductive thinking raised by selected questions (Figure 6).

### 4.2. Guidelines for Leishmania Extracellular Vesicle Research

The International Society for Extracellular Vesicles (ISEV) proposed a gold standard for the isolation and analysis of extracellular vesicles [19,87,88]. However, studies in Medical Parasitology still lack a consensus on the ultrasensitive detection of the specific biomarkers of different extracellular vesicle subtypes, which may originate endosomes, exosomes, derivatives of the plasma membrane or ectosomes, failing to define their specific biogenesis pathways reliably [18,19,91]. Given this, the standardization of research criteria should be a priority for future studies [19,87,88,91]. Based on this approach, we would suggest the following initial guidelines for the production of parasite cells in serum-free culture medium based on standardized research parameters (Table 2) [87,88,91]:

1. Optimization of the cell culture and harvest conditions; (b) cell culture and exosome production, and the culture of adherent cells to confluence (e.g., RPMI 1640 medium with 10% ultrapure FBS, which provides the highest level of exosome depletion, 1 mM sodium-pyruvate in 225 cm^2^ cell culture bottles at 37 °C with 5% CO_2_); (c) removal of the medium from the confluent cells through the addition of 50 mL of fresh medium; (d) after 3 days, remove the cell-conditioned medium, with two centrifugation steps (300× *g* for 10 min at 2–8 °C and 2000× *g* for 30 min at 2–80 °C) prior to pre-enrichment [87].
2. Test for exosome release; (b) depending on the cell type and the production efficiency, it may be possible to extract exosomes from the solution directly without any pre-enrichment step (e.g., flow analysis); (c) this is very useful to verify the efficiency of the exosome release by the cell and determine the correct time for exosome harvesting; (d) use of Dynabeads™ (CD9, CD63 or CD81) as exosomal markers, first in the host cell first and then in the *L*EVs (e.g., flow analysis) [87].
3. Confirmation of the characteristics of the vesicles using multiple methods—RNA and protein detection, EM, specific markers found in non-exosome vesicles (e.g., ER, Golgi, etc.) [87,88,91,113].
4. Key questions asked by the extracellular research community; (b) current recommendations: estimating the size distribution using, e.g., Nano Tracking Analysis (NTA); (c) combining NTA with Transmission Electron Microscopy (TEM); (d) verifying the presence of miRNA/mRNA; (e) using Western Blotting (WB) to target lipid-bound targets such as tetraspanins (3 different types), cytosolic proteins, such as TSG101, annexins, and rabs, the absence or under-representation of the ER (e.g., hsp90B1, calnexin), Golgi (GM130), mitochondrion (cytC) or nucleus (histones); (f) flow cytometry can be used easily to support the WB data (e.g., for membrane-anchored targets such as tetraspanins) using Dynabeads™ as the solid support, which can be introduced and detected in the flow instrument [91].
5. Consider the question “can the exosome harvest be used directly or do I need to pre-enrich?”; (b) the flow signal you will obtain may vary from very low/absent to signals that are sufficient for further analysis. Depending on this signal, the researcher may decide to continue to direct isolation with the Dynabeads™, give that they are compatible with many different types of downstream applications [91].

These steps should contribute to the more reliable identification of the specific functions of the *L*EVs, or the definition of their subtypes, and extend the potential data beyond the simple description of function to crude, potentially contaminated, and heterogeneous isolates [87,88,91].

## 5. Discussion

Extracellular vesicles are naturally secreted by all cells, including those of *Leishmania* species, and can be found in all body fluids, where they are involved in a range of processes, such as the eradication of obsolete molecules, the dissemination of oncogenes from cancer cells, and cell-to-cell communication, including the spread of pathogens [76,115,122,123]. Recently described, high performance methods for the isolation of products have been applied to research on protozoan extracellular vesicles, although no consensus has been reached on the ultrasensitive detection of the specific biomarkers of different extracellular vesicle subtypes, which may originate endosomes, exosomes, derivatives of the plasma membrane or ectosomes, failing to define their specific biogenesis pathways reliably [18,91]. According to Olajide and Cai [8], combining filtration or concentration with ultracentrifugation through a sucrose gradient cushion should guarantee the retention of intact membrane vesicles. A commercial exosome purification kit, which can be used to precipitate a wider or more restricted range of vesicles has also been used for the isolation of pathogenic protozoan extracellular vesicles, although this approach has yet to be validated categorically. These same authors concluded that the populations of vesicles obtained by differential centrifugation and ultracentrifugation will most often provide a mixed population of extracellular vesicles with soluble proteins not associated with vesicles, so that the recovery of the extracellular vesicles following the filtration of the culture may not provide a complete picture of the extracellular products of the parasite [8]. They also observed that size exclusion chromatography has been advocated in situations in which a higher yield of extracellular vesicles is required, and that the method adopted to isolate the extracellular vesicles has a considerable influence on the proteomic profile of the pathogenic protozoan, which further compounds the difficulty of extrapolating the findings between different proteomic studies [8]. The authors also conclude that physicochemical stimuli may play an important role in the content and function of isolated extracellular vesicles [8]. Despite all these considerations, the research has, in general, focused primarily on the description of the different clinical forms of *L*EV, in particular, in the visceral strains and in the proteomics of *L.* (*L.*) *mexicana* under the same conditions, which has provided valuable information on how polymorphisms in the *L*EV proteins may affect the cell-to-cell interactions between parasites and the host-parasite or the leishmanicidal activity [4,12,14,35,124]. Even so, comparative analyses of the reproducible isolation and the description of *L*EVs from procyclic and metacyclic-like in vitro cultures of a wider range of *Leishmania* species are still scarce [76,116,122]. New technologies such as proteomics have advanced existing knowledge on the differential expression of virulence factors in the different parasite stages, and the functional activity induced by the *L*EVs released by *L.* (*L.*) *infantum*, although the comparative proteomics of *L*EV production during the in vivo parasite cycle are still lacking [4,34,35,85,86]. In vitro *L*EVs released from *L.* (*L.*) *donovani* have been used to provoke functional responses in target cell assays, which have demonstrated that dendritic cells derived from monocytes increased TNF-α, IL-6, and IL-8, reduced CD80, CD86, HLA-DR, and also increased IFN-γ, IL-10, IL-17. Other in vitro assays using *L*EVs released by *L.* (*L.*) *donovani* showed that the splenocytes higher IFN-γ, IL-4(CD4) T cells and the spleen lymph node, lower IFN-γ (CD4 T cells) and Foxp3 [8]. Findings from other in vitro studies have shown that *L*EVs released by *L.* (*L.*) *infantum* induce IL-10 in humans cells and reduced IL-18 using monocytes, while *L*EVs released by *L.* (*L.*) *major* induced IL-17A, IL-4, IL-23, and IFN-y in the lymph nodes and *L*EVs released by *L.* (*L.*) *amazonensis* increased IL-6 and IL-10 using macrophages, but increased IL-6 and decreased IL-10 through B-1 cells [8]. Virulence factors secreted by *Leishmania* interfere with intracellular macrophage signaling, provoking the activity of different cytokines [40]. Hypothetical virulence factors may then become a part of the specific *L*EVs molecular signature (proteins and nucleic acids with no ability to replicate) during the evolution of the parasite, which affects the host immune system, although no significant differences were found among strains in the number of proteins in from the secretome [3,113]. Regardless of the parasitic form, *Leishmania* secretes *L*EVs containing virulence factors. Studies of the promastigotes of *L.* (*L.*) *infantum* and *L.* (*L.*) *major* in the midgut of infected sand flies identified the *L*EVs egested by the flies, which contained varying amounts of virulence factor GP63 [80]. The considerable biological similarities between *Leishmania* and other trypanosomatid pathogens imply that insights obtained into the biology of the *L*EVs may be equally relevant to studies of both other trypanosomatids and other protozoan parasites, such as *Toxoplasma* and *Plasmodium* [75,76,85].

Comparative studies between cutaneous and visceral species must also be advanced in order to assess their in vitro responses and their subtle differences. A comparative experimental evaluation of the interactions of the *L*EVs (highest yield rate around 40 nm. Enhanced graphic peaks also observed at 60–70 nm and 150–160 nm) released by the promastigotes and amastigotes of cutaneous and visceral *Leishmania* species with mouse macrophages was proposed in the original findings of Gabriel et al. (2017) [82]. These *L*EVs had the capacity to modulate the activity of the macrophage in the M2 stage, which favors the survival of the parasite. The *L*EVs of (*L.* (*L.*) *infantum*, *L.* (*L*.) *amazonensis*, *L.* (*Viannia*) *shawi*, and *L.* (*V*.) *guyanensis* increased significantly the production of urea, with the exception of those of the amastigotes of *L.* (*L.*) *infantum* [82]. However, these *L*EVs also inhibited nitric oxide (NO) significantly. These results indicate that the *L*EVs produced by both morphotypes of the parasite of the different *Leishmania* species responsible for visceral and cutaneous leishmaniasis in humans drive the macrophages for an anti-inflammatory phenotype [82].

The *L*EVs play an important role in infectivity and modulate the host’s immune response through the *Leishmania* promastigotes and amastigotes [3,5,34]. While *L*EVs have been found to play an active role in the mammalian host, little is understood about their effects on the sand fly, or how they might affect the infection of this insect by the parasite [14,40,75,76,85]. Overall, then, the parasites are distinguished by their morphology, environmental, and nutritional requirements, and by their in vitro stimulus, metabolism, motility, growth rate, and multiplication in the host or the culture medium and, more recently, by the molecular expression of specific *L*EVs [3,8,34]. For this reason, the isolation of *L*EVs from different developmental stages may provide important insights into the possible alterations they undergo over the course of the parasite’s life cycle [14,40,75,76,85]. Exosomal protein groups (1) transmembrane or GPI-anchored proteins associated with the plasma membrane and/or endosomes; (2) cytosolic proteins recovered from *L*EVs; (3) major components of non-*L*EVs co-isolated structures; (4) transmembrane, lipid-bound, and soluble proteins associated with intracellular compartments other than the PM/endosomes; (5) secreted proteins recovered by *L*EVs) found in both parasite phases should be the focus of future studies that assess potential targets for intercellular modulation [14,75,76,85,88]. These findings on the composition of the *L*EV proteome raise many questions with regard to their function and provide compelling evidence that the *L*EVs play an active molecular role in the parasite’s development in both the vertebrate and the invertebrate hosts in their specific pathways. In addition, the parasite morphology associated with the biological functions linked to the modulation of the immune responses of the host to the *L*EVs provides an important potential field for the investigation of virulence factors and the mechanisms for survival, and their broad immunological roles within the cell in the vertebrate host, possibly with parallel studies in the invertebrate host [3,34,82]. This may be a lucrative starting point for the development of new preventive and therapeutic strategies with more efficient pharmacological resources to combat *Leishmania*, which continues to be difficult to prevent and treat, given that the available drugs are not only toxic, but are also not completely effective. Some studies of the role of *Leishmania* proteins have identified protein kinases from the casein kinase family, Aurora, and other kinase families as possible pathways for the development of new drugs [125]. Concomitantly, comparative metabolomic and proteomic analyses may help to consolidate and expand the existing knowledge on the role of *L*EVs (Table S1). Clearly, many alternative hypotheses are still to be tested in the broad context of *L*EVs, and more *L*EV studies are needed, given that one of the fundamental points for the control of endemic parasitic diseases is the reliable understanding of the mechanisms that underpin the pathogen-host interaction [3,34,82].

## 6. Conclusions

Overall, then, this review compiles evidence showing that *Leishmania*, like other eukaryotes, uses extracellular vesicles as mechanisms of infection and for the modulation of the immune response of the host, with a primary focus on the establishment of infection in humans and other animals. Despite the many recent advances, considerable gaps remain in our knowledge of the biological mechanisms and functional conditions involved in the release of *L*EVs, and how they affect the parasite host interaction [40,41,42,43,44,45,46,47,48,49,50,51,52,53,54,55,56,57,58,59,60,61,62,63,64,65,66,67,68,69,70,71,72,73,74,75,76,77,78,79,80,81,82,83,84,85,86,87,88,89,90,91]. The recent research does point to a nano-drug delivery system as a potentially important step forward for the improvement of anti-leishmanial therapy [16]. Similar pathway-oriented analyses should also be used to identify the targets of *L*EVs *in vivo*. Future molecular research in this field of Parasitology will be crucial to the eventual control of leishmaniasis worldwide.

## Figures and Tables

**Table 1:** Old and New World *Leishmania* species considered a potential source of animal and human infection, their main characteristics and what was published about their extracellular vesicles [8,20,24,26,27,121]. *** No additional information available.

Species	Geographic Distribution	Clinical Disease in Humans	Hosts	Experimental Activatory Stimuli	*L*EVs Isolation Methods	*L*EVs type and Sub-Cellular Origin	Size (Mean/ Range)	Major *L*EVs Content	Method of *L*EVs Analysis
Complex			Vector: Phlebotomine sand fly species			Microparticles,			
*L.* (*L.*) *donovani*	Central and southern Sudan, Northern Kenya, south-east Ethiopia, Uganda, Chad, Yemen, North-east India, Bangladesh, Terai region of Nepal, Buthan, China	VL, PKDL	Infect wild and synathropic rodents, wild felids, viverrids. Cats (*Felis silvestris catus*)? Presumably zoonotic, but reservoir host uncertain/unknown. Human (peridomestic) and Herpestids Mongooses (zoonotic)	Neutral and acid medium; RPMI with HEPES, MES;	4, 2, 7	(Exosomes, vesicles);	30–70 nm	HSP100, 90, 70.4, GP63, histone, chaperonin, proteins; TESA, trans-sialidases, protease transport, metabolic protein	Immunofluorescence (IF) and EM; Proteomic Analysis
						*L.* (*L.*) *donovani* HSP100/and			
						Wildtype (Exosomes)			
						From PM, FP, phagolysosome			
*L.* (*L.*) *infantum* *(syn. of L. L. chagasi)*	Central and western Mediterranean basin, both Europe and North Africa Through Mediterranean basin to Iran, South west Asia, China Central and South America	VL, CL (zoonotic), Infantile visceral leishmaniasis, AIDS associated leishmaniasis	Vectors: Several species of Phlebotomus and Lutzomyia sandflies Infect wild canids, synanthropic rodents, cats and humans Dogs are reservoir hosts (viscerocutaneous leishmaniasis) Landscape peridomestic	Miltefosine/apoptosis indicers, G418; Insect gut; RPMI pepton Yeast; Exo-free medium [77,82,114]	5,3; 11; 1,4; 8	Vesicles, Exosome-like Vesicles, Exosome Vesicles	30–100 nm; 50–120 nm; 122 + −56 nm; 30–450 nm	GP63, ribossomal protein, hsp70 elongation factor-1 α, β tubulin, β-fructofuranosidases; HSP70, HSP83/90, Acetylcholinesterase; GP63, calpan-like cysteine peptidase, HSP70, tryparedoxin peroxidase surface antigen protein; Nucleic acids and proteins (protein levels ranged from 40 ± 76 mg.mL^−1^, RNA concentration reached mean values of 90 ± 277 ng.µL^−1^ and DNA of 85 ± 377 ng.µL^−1^)	Experimental infection [77,82,114]
						From membrane surface, FP, MVB; Extracellular vesicles, nanovesicles, exosomes [77,82,114]	115 + −65 nm		
*L. (L.) major*	Sub-Saharan Africa, Yemen, North Africa, Middle East. South-west Asia, from Algeria to Saudi Arabia. Iran and neighbours, Pakistan, Northwest India. Central Asia from Iran to Uzbekistan, west Africa to Kenya, Sahel belt	CL, Oriental sore (wet form)	Vector: Phlebotomine sand fly species *(Phlebotomus*) Infect numerous desert mammals, wild rodents, cats, dogs and humans Reservoir hosts: Fat sand-rat *Psammomys obesus* (epidemic maintained by *Meriones shawi*), great gerbil *Rhombomys opimus*, regional gerbils and rodents Relative importance of different hosts to be determined	Neutral and acid medium; Insect gut;	11; 4	Microparticles, (Exosomes, vesicles); Exosome-like Vesicles	30–70 nm; 50–120 nm	GP63, calpan-like cysteine peptidase, HSP70, tryparedoxin peroxidase surface antigen protein; TESA, trans-sialidases, protease transport, metabolic protein	Proteomic Analysis
						From membrane surface, FP, MVB, PM, phagolysosome			
*L. (L.) amazonensis* *(syn. of L. L. garnhami)*	South America, mostly North of the Amazon, East of Andes	CL, DCL, MCL	Vector: Phlebotomine sand fly species (*Lutzomyia)* Infect terrestrial forest rodents, marsupials, procyonids, wild canids, edentates, sciuridi and humans (sylvatic) Terrestrial rodents and marsupials, *Proechymis guyanensis* and *Proechymis cuvieri* (reservoir hosts)	RPMI/20% glucose; Exo-free medium [77,78,79,80,81,82,83,84,85,86,87,88,89,90,91,92,93,94,95,96,97,98,99,100,101,102,103,104,105,106,107,108,109,110,111,112,113,114]	2,4; 1;8	*L. (L.) amazonensis* P (-M2269) (Evs) whole body; Extracellular vesicles, nanovesicles, exosomes [77,82,114]	180 nm; 30–450 nm	GP63, LPG; Nucleic acids and proteins (protein levels ranged from 40 ± 76 mg·mL^−1^, RNA concentration reached mean values of 90 ± 277 ng.µL^−1^ and DNA of 85 ± 377 ng.µL^−1^)	Experimental infection [77,82,114]
*L. (L.) mexicana* *(syn. of L. L. pifanoi)*	Southern USA (Texas), Central and South America, East of Andes	CL, DCL, Chiclero ulcer	Vector: Phlebotomine sand fly species (*Lutzomyia*) Infect rodents, edentates, marsupials, wild canids and humans (sylvatic) Marsupials and terrestrial rodents: *Ototylomys phyllotis* and *Neotoma micropus* (reservoir hosts)	Neutral and acid medium	4	Microparticles,	30–70 nm	TESA, trans-sialidases, protease transport, metabolic protein	Proteomic Analysis
						(Exosomes, vesicles)			
						From PM, FP, phagolysosome			
*L. (L.) shawi*	Brazilian Amazon Region	CL	Vector: Phlebotomine sand fly species (*Lutzomyia)* Infect monkeys: *Cebus apella*, *Chiropotes satanas*; edentates: *Choloepus didactylus*, *Bradypus tridactylus*; procyonids: *Nasua nasua*; humans (sylvatic) Main host uncertain	Exo-free medium [77,82,114]	1; 8	Extracellular vesicles, nanovesicles, exosomes [77,82,114]	30–450 nm	Nucleic acids and proteins (protein levels ranged from 40 ± 76 mg.mL^−1^, RNA concentration reached mean values of 90 ± 277 ng.µL^−1^ and DNA of 85 ± 377 ng.µL^−1^)	Experimental infection [77,82,114]
*L. (V.) braziliensis*	Central & South America	CL, MCL	Vector: Phlebotomine sand fly species (*Lutzomyia)* Infect wild and synanthropic rodents, marsupials: *Didelphis marsupialis* and others numerous forest animals; dogs (CL), cats, horses, donkeys Humans. (peridomestc and sylvatic) Presumably zoonotic, but reservoir host unknown Rodents? Marsupials? Dogs?	199 medium (Gibco, Life Technologies Brand, Grand Island, NY, USA) supplemented with 4.2 mM sodium bicarbonate, 4.2 mM HEPES, 1 mM adenine, 5 μg/mL hemin (bovine type I) (Sigma, St. Louis, MO, USA) and 10% fetal calf serum (FCS) (Gibco, Carlsbad, CA, USA) [1]	5	Extracellular vesicles	***	***	Proteomic Analysis
*L. (V.) guyanensis*	South America, East of Andes, Guyanas	CL, MCL	Vector: Phlebotomine sand fly species (*Lutzomyia)* Infect wild rodents, edentates: *Choloepus didactylus*, *Tamandua tetradactyla*; marsupials, humans (zoonotic) Sloth *Choloepus didactylus* (reservoir host) and probably other animals (sylvatic)	Exo-free medium [77,82,114]	1; 8	Extracellular vesicles, nanovesicles, exosomes [77,82,114]	30–450 nm	Nucleic acids and proteins (protein levels ranged from 40 ± 76 mg·ml^−1^, RNA concentration reached mean values of 90 ± 277 ng.µL^−1^ and DNA of 85 ± 377 ng.µL^−1^)	Experimental infection [77,82,114]

1. Centrifugation 2. Filtration, 3. Concentration by ultrafiltration/high molecular weight cut-off filter, 4. Sequential/serial centrifugation 5. Ultracentrifugation, 6. Buoyant density on Optiprep gradient fractionation, 7. Buoyant density on sucrose gradient fractionation, 8. Precipitation by exo-prep kit, 9. Gel exclusion chromatography, 10. Size exclusion chromatography, 11. Dissection/Suspension in PBS FP, flagellar pocket, PM, plasma membrane [8].

**Table 2:** Checklist for *Leishmania* extracellular vesicles (*L*EVs) research [87,88].

Parameters	Mandatory Steps (Standardized)	Mandatory if Applicable	Encouraged Steps
**Nomenclature**	(a) We propose to use the generic term *Leishmania* extracellular vesicles (*L*EVs): With demonstration of extracellular (no intact parasites) and vesicular nature per these characterization and function guidelines	No additional information available	1. Use the generic term *Leishmania* extracellular vesicles (*L*EVs) + specification (considering size, density, others)
	(b) Generic term *Leishmania* extracellular particles (*L*EPs): No intact parasites but MISEV guidelines not satisfied		1. Use a specific term for subcellular origin: *Leishmania* Ectosomes, microparticles, microvesicles (from plasma membrane), exosomes (from endosomes), with demonstration of the subcellular origin 2. Use other specific term: with definition of specific criteria
**Collection and pre-processing culture conditioned medium (CM)** **General cell characterization medium used before and during collection (additives, serum, other)**	(a) Nature and size of culture vessels, and volume of medium during conditioning	1. Exact protocol for depletion of *L*EVs/*L*EPs from additives in collection medium	No additional information available
	(b) Number of parasites/mL, or/surface area and % of live/dead cells at time of collection (or at time of seeding with estimation at time of collection) (c) Frequency and interval of CM harvest	2. Specific culture conditions (treatment, % O_2_, others) before and during collection	
**Collection and pre-processing** **Storage and recovery**	(a) Storage and recovery (e.g., thawing) of culture medium, before *L*EVs isolation (storage temperature, vessel, time; method of thawing or other sample preparation)	No additional information available	No additional information available
	(b) Storage and recovery of *L*EVs after isolation (temperature, vessel, time, additive(s) others)		
***L*EVs separation and concentration** **Experimental details of the method**	No additional information available	(a) Centrifugation: reference number of tube(s), rotor(s), adjusted k factor(s) of each centrifugation step (= time + speed + rotor, volume/density of centrifugation conditions), temperature, brake settings	No additional information available
		(b) Density gradient: nature of matrix, method of generating gradient, reference (and size) of tubes, bottom-up (sample at bottom, high density) or top-bottom (sample on top, low density), centrifugation speed and time (with brake specified), method and volume of fraction recovery	
		(c) Chromatography: matrix (nature, pore size, others), loaded sample volume, fraction volume, number	
		(d) Precipitation: references, ratio vol/vol or weight/vol fluid, time/temperature of incubation, time/speed/temperature of centrifugation	
		(e) Filtration: reference of filter type (= nature of membrane, pore size, others, time and speed of centrifugation, volume before/after (in case of concentration)	
		(f) Antibody-based: reference of antibodies, mass Ab/amount of *L*EVs, nature of Ab carrier (bead, surface) and amount of Ab/carrier surface	
		Other: all necessary details to allow replication	
		Additional step(s) to concentrate	
		Additional step(s) to wash matrix and/or sample	
***L*EVs separation and concentration** **Specify category of the chosen *L*EVs separation/concentration method**	No additional information available	No additional information available	1. High recovery, low specificity = mixed *L*EVs and non-*L*EVs components
			2. Intermediate recovery, intermediate specificity = mixed *L*EVs with limited non-*L*EVs components
			3. Low recovery, high specificity = subtype(s) of *L*EVs with as little non- *L*EVs as possible
			4. High recovery, high specificity = subtype(s) of *L*EVs with as little non-*L*EVs as possible
*L*EVs characterization (Quantification)	(a) Volume of fluid, and/or cell number, and/or tissue mass used to isolate *L*EVs	No additional information available	No additional information available
	(b) Global quantification by at least 2 methods: protein amount, particle number, lipid amount, expressed per volume of initial fluid or number of producing cells/mass of tissue Ratio of the 2 quantification figures		
*L*EVs characterization (Global characterization)	(a) Transmembrane or GPI-anchored protein localized in cells/parasites at plasma membrane or endosomes	1. Presence of proteins associated with compartments other than plasma membrane or endosomes	Topology of the relevant functional components
	(b) Cytosolic protein with membrane-binding or-association capacity Assessment of presence/absence of expected contaminants	2. Presence of soluble secreted proteins and their likely transmembrane ligands	
***L*EVs characterization** **Single *L*EVs characterization**	(a) Images of single *L*EVs by wide-field and close-up: e.g., EM, scanning probe microscopy, superresolution fluorescence microscopy	No additional information available	No additional information available
	(b) Non-image-based method analysing large numbers of single *L*EVs: NTA (Nanoparticle tracking analysis), Tunable resistive pulse sensing (TRPS), Fluorescence Correlation Spectroscopy (FCS), high-resolution flow cytometry, multi-angle light-scattering, Raman spectroscopy, along with others.		
**Functional studies**	(a) Dose-response assessment	No additional information available	1. Quantitative comparison of activity of *L*EVs subtypes (if subtype-specific function claimed)
	(b) Negative control = nonconditioned medium, biofluid/tissue from control donors, as applicable		
	(c) Quantitative comparison of functional activity of total fluid, vs. *L*EVs-depleted fluid, vs. *L*EVs (after high recovery/low specificity separation)		2. Extent of functional activity in the absence of contact between *L*EVs donor and *L*EVs recipient
	(d) Quantitative comparison of functional activity of *L*EVs vs. other *L*EPs/fractions after low recovery/high specificity separation		
**Reporting**	Submission of data (proteomic, sequencing, others) to relevant public, curated databases or open-access repositories	Temper *L*EVs-specific claims when MISEV requirements cannot be entirely satisfied	1. Submission of methodologic details to *L*EVs-TRACK (evtrack.org) with EVs-TRACK number provided (strongly encouraged)
			2. Data submission to *L*EVs -specific databases (e.g., EVpedia, Vesiclepedia, exRNA atlas, Eco Carta, exoRBase,)

## Data Availability

The data presented in this study are available on request from the corresponding author (Á.M.G.). The data are not publicly available due to confidentiality.

## Supplementary Materials

The following are available online at https://www.mdpi.com/2076-2607/9/10/2081/s1, Table S1: Old and New World Leishmania species considered a potential source of animal and human infection without LEVs research previously considered or tested (*** open field for future research).
