# Supplementary material for: Leishmania 360°: Guidelines for Exosomal Research
Source: Microorganisms. 2021 Oct 2;9(10):2081. doi: 10.3390/microorganisms9102081 (PMC8537887; doi:10.3390/microorganisms9102081)
Supplement: Supplementary file 1 [file microorganisms-09-02081-s001.zip › microorganisms-1385797-supplementary.pdf]

microorganisms

Supplementary Table S1. Old and New World *Leishmania* species considered a potential source of animal and human infection without *L*EVs research previously considered or tested (*** open field for future research) [8,20,24,26,27,121].

Species	Geographic Distribution	Clinical Disease in Humans	Hosts	Experimental Activatory Stimuli	*L*EVs Isolation Methods	*L*EVs type and Sub-Cellular Origin	Size (Mean/ Range)	Major *L*EVs Content	Method of *L*EVs Analysis
L.* (*M.*) *enriettii	Present in Brazil	Human infections undetected. Emerging as new human pathogens, causing VL and CL in HIV⁺ and HIV⁻ patients<br>Prevalence of undiagnosed cases and asymptomatic infections?	Life cycle remains poorly understood<br>The identity of vectors responsible for transmission has not been confirmed for any species yet<br>Infect domestic Guinea pigs.<br>Detected in wildlife kept in captivity.	***	***	***	***	***	***
L.* (*M.*) *macropodum	Present in Australia	Human infections undetected. Prevalence of undiagnosed cases and asymptomatic infections?	Life cycle remains poorly understood<br>Vector: (possibly, Phlebotomine sand fly species and biting midges (*Forciponyia* sp.)<br>Detected in wildlife kept in captivity (Kangaroo species)	***	***	***	***	***	***
***L.* (*M.*) *martiniquensis*,**	Present in Martinique island, Florida,	CL, DCL, VL Prevalence of undiagnosed cases and	Life cycle remains	***	***	***	***	***	***

	Switzerland, Germany and south-east Asia	asymptomatic infections?<br>VL in BALB/c mice	poorly understood<br>Vectors (?)<br>Detected in human and domestic animals						
**L. (*M.*) *orientalis***	Present in south-east Asia	CL, DCL, VL<br>Prevalence of undiagnosed cases and asymptomatic infections?	Life cycle remains poorly understood<br>The identity of vectors responsible for transmission has not been confirmed for any species yet.<br>Detected in humans.	***	***	***	***	***	***
***L.* (*M.*) sp.** Ghana that is still formally undescribed	Present in Africa	CL<br>Prevalence of undiagnosed cases and asymptomatic infections?	Life cycle remains poorly understood<br>The identity of vectors responsible for transmission has not been confirmed for any species yet.<br>Detected in human.	***	***	***	***	***	***
L. (L.) aethiopica	Highlands of Ethiopia and Kenya, highlands of south-west Ethiopia	CL, DCL, MCL, Chronic oriental sore Zoonotic	Vector: Phlebotomine sand fly species (*Phlebotomus*)<br>Infect wild rodents.	***	***	***	***	***	***

			Rock hyraxes, *Procavia capensis*, *Heterohyrax brucei* (reservoir hosts)						
L. (L.) gerbilli	Eastern Russia, Mongolia	Human infection unknown	Vector: Phlebotomine sand fly species (*Phlebotomus*)<br>Great gerbil *Rhombomys opimus* (reservoir host)	***	***	***	***	***	***
L. (L.) turanica	Central Asia	Human infection unknown	Vector: Phlebotomine sand fly species (*Phlebotomus*) Great gerbil *Rhombomys opimus* (reservoir host)	***	***	***	***	***	***
L. (L.) arabica	Saudi Arabia	Human infection unknown	Vector: Phlebotomine sand fly species (*Phlebotomus*)<br>Fat sand-rat *Psammomys obesus* (reservoir host)	***	***	***	***	***	***
L. (L.) tropica	Central to south-west Asia, North, equatorial and southern Africa, Middle east, Iran, Afghanistan, Sub-Saharan Africa, Kenya, Ethiopia and Namibia. Mediterranean	CL, VL Oriental sore (dry form), leishmaniasis recidivans	Vector: Phlebotomine sand fly species (*Phlebotomus*)<br>Infect wild rodents and wild canids? cats? dogs (CL), and humans.<br>Humans are reservoir hosts (peridomestic).	***	***	***	***	***	***

			Probably rock hyraxes and *Procavia capensis*.						
L. (L.) venezuelensis	Northern Venezuela, in the States of Lara and Yaracuy, Dominican Republic	CL, DCL	Vector: Unknown (*Lutzomyia*?)<br><br>Infect *d*omestic cats? humans? wild and synanthropic rodents? Presumably zoonotic and sylvatic, but reservoir host unknown.	***	***	***	***	***	***
L. (V.) colombiensis	Colombia, Panama, Colombia, Venezuela, forests of Brazil and Peruvian lowlands, other Latin American countries	CL, VL	Vector: Phlebotomine sand fly species (*Lutzomyia)*<br><br>Infect sloths *Choloepus hoffmanni* (reservoir host) and humans (sylvatic)	***	***	***	***	***	***
L. (V.) lainsoni	Forested areas of Northern Pará Brazil, Guianas, Peru and Bolivia	CL	Vector: Phlebotomine sand fly species (*Lutzomyia)*<br><br>*Cuniculus paca* (reservoir host)<br><br>Presumably zoonotic and sylvatic	***	***	***	***	***	***
L. (V.) lindenbergi	Degraded forest in Belém, Pará, Brazil	CL	Vector: Phlebotomine sand fly species (*Lutzomyia)*<br><br>Infect humans. It is suspected that the wild animal	***	***	***	***	***	***

			reservoirs are probably terrestrial						
L. (V.) naiffi	States of Pará and Amazonas (Brazil), French Guyana, Panama	CL	Vector: Phlebotomine sand fly species (*Lutzomyia)*<br>Nine- banded armadillo *Dasypus novemcinctus* (reservoir host)<br>Presumably zoonotic and sylvatic.	***	***	***	***	***	***
L. (V.) panamensis	South and Central America, West of Andes, northern Venezuela, Panama	CL, MCL	Vector: Phlebotomine sand fly species (*Lutzomyia)*<br>Infect Synanthropic and Neotropical rodents, edentates, marsupials, procyonids, erethizontides, monkeys, hunting dogs and humans (primary forest, zoonotic).<br>Sloth *Choloepus hoffmanni* and probably other animals (reservoir host)	***	***	***	***	***	***
L. (V.) peruviana	South America, Arid valleys of the western Peruvian Andes	CL, MCL	Vector: Phlebotomine sand fly species (*Lutzomyia)*	***	***	***	***	***	***

	Infect dogs (CL) and humans.	
	Reservoir hosts: Rodents (*Phyllotis andinum* ?), marsupials (*Didelphis marsupialis* ?) and dogs (zoonotic).<br><br>Transmission is thought mainly to be dependent on humans (peridomestic and sylvatic)	

1. Centrifugation 2. Filtration, 3. Concentration by ultrafiltration/high molecular weight cut-off filter, 4. Sequential/serial centrifugation 5. Ultracentrifugation, 6. Buoyant density on Optiprep gradient fractionation, 7. Buoyant density on sucrose gradient fractionation, 8. Precipitation by exo-prep kit, 9. Gel exclusion chromatography, 10. Size exclusion chromatography, 11. Dissection/Suspension in PBS FP, flagellar pocket, PM, plasma membrane [8]
